# Generation of sarconoids from angiosarcoma patients as a systematic-based rational approach to treatment

**DOI:** 10.1186/s13045-024-01556-3

**Published:** 2024-05-20

**Authors:** Da Jung Jung, Jae Hee Byeon, Young Chul Kim, Woo Shik Jeong, Jong-Woo Choi, Gi Seok Jeong

**Affiliations:** 1 Biomedical Engineering Research Center, Asan Institute for Life Sciences, Seoul, South Korea; 2 Department of Covergence Medicine, College of Medicine, University of Ulsan, Seoul, South Korea; 3 Department of Plastic and Reconstructive Surgery, Asan Medical Center, Seoul, South Korea; 4 Department of Plastic and Reconstructive Surgery, College of Medicine, University of Ulsan, Seoul, South Korea; 5 Department of Biomedical Engineering, College of Medicine, University of Ulsan, Seoul, South Korea

**Keywords:** Angiosarcoma, Organoids, PROCR, Translational research, Drug screening platform

## Abstract

Angiosarcoma is a rare subtype of malignant neoplasm originating from vascular or lymphatic endothelial cells; its low incidence has posed significant challenges for comprehensive investigations into its pathogenic mechanisms and the development of innovative treatment modalities through in vitro and in vivo models. Recent endeavors spearheaded by patient-partnered research initiatives have aimed to elucidate the intricacies of angiosarcomas by leveraging biological omics approaches, with the overarching objective of enhancing prognostic indicators and therapeutic options for this uncommon pathology. To bridge the gap between preclinical research and translational applications, we engineered angiosarcoma-derived organoids from surgically resected primary tumors, hereafter referred to as “sarconoids,” as a proof-of-concept model. A novel protocol for the establishment of these sarconoids has been developed and validated. To ensure that the sarconoids faithfully recapitulate the heterogeneity and complexities of the patients’ original tumors, including transcriptomic signatures, cell-type specificity, and morphological traits, exhaustive histological and transcriptomic analyses were conducted. Subsequently, we expanded the scope of our study to include an evaluation of a sarconoid-based drug screening platform; for this purpose, a drug library (AOD IX), supplied by the National Cancer Institute’s Developmental Therapeutics Program, was screened using 96-well plates. Our findings suggest that sarconoids can be reliably generated from angiosarcoma patient-derived tissues and can serve as accurate models for evaluating therapeutic responses, thereby holding far-reaching implications for translational research and clinical applications aimed at advancing our understanding and treatment of angiosarcoma.

**Supplementary Information:**

The online version contains supplementary material available at 10.1186/s13045-024-01556-3.

## To the editor

Patient-derived cancer organoids have become increasingly pivotal in pre-clinical and translational cancer research, having been generated from a diverse array of human cancer tissues [1, 2]. Notably, most extant human cancer organoid models are derived from ectodermal or endodermal epithelial progenitors, and, to our knowledge, none have originated from mesenchymal or endothelial tissues [3, 4].

Angiosarcoma is a rare yet aggressive mesenchymal tumor of endothelial origin [5]. There are several specific challenges associated with the clinical development of therapeutics for this disease, such as absence of prior clinical studies, and a lack of information on disease mechanisms and progression [6, 7].

**Fig. 1:**
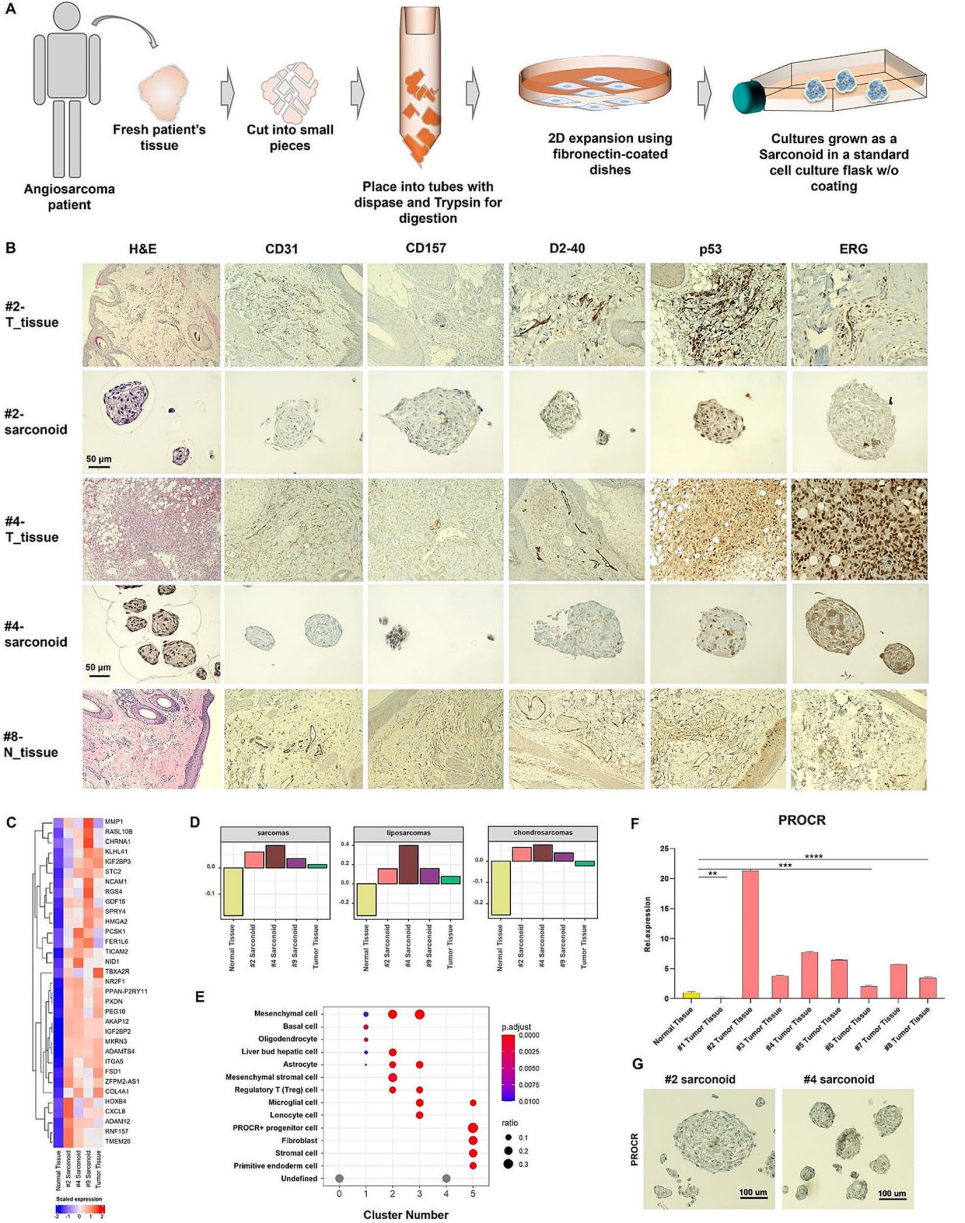
Establishment and characterization of sarconoids derived from angiosarcoma patients. (**a**) Schematic illustration of the workflow for the generation of sarconoids. (**b**) Histopathological staining of vascular neoplasm markers (CD31, CD157, ERG, and p53) and a lymphatic marker (D2-40) in primary resected angiosarcoma tumors and their corresponding sarconoids; scale bars, 50 μm. (**c**) Heat map of the scaled FPKM values of representative gene sets significantly upregulated in the patient-derived sarconoids and tumor tissue. (**d**) Gene set variation analysis (GSVA)-based subtyping of normal tissue, patient #2 tumor tissue, and patient #2-, #4 and #9-sarconoids against published gene sets (GeneRIF Biological Term Annotations), among the sarcoma subtypes. (**e**) Dot plot of the mean expression of canonical marker genes using the CellMarker database for 14 major lineages from each patient #2-sarconoid cluster, as indicated. (**f**) Quantification of PROCR expression by qRT-PCR in normal and cancer tissues (*n* = 3). An unpaired t-test was used; *p < 0.05, **p < 0.01,***p < 0.001,****p < 0.0001. (**g**) IHC assays of serial sections from patient #2- and patient #4-sarconoids for PROCR expression. Scale bars, 100 μm

In this study, we propose a methodology to develop personalized angiosarcoma organoid models, termed “sarconoids,” for biological characterization and high-throughput drug screenings. To identify FDA-approved drugs amenable to repurposing for individualized treatment of rare diseases, we generated sarconoids using surgically resected angiosarcoma specimens (Fig. 1a, Fig. S1a). Immunohistochemical analyses and quantitative real-time PCR (qPCR) analyses revealed elevated expression levels of various vascular and mesenchymal markers in both primary tumors and tumor-derived sarconoids, as compared to normal tissues (Fig. 1b, Fig. S1c, Fig S2a-c). To evaluate the angiogenic potential of our sarconoids, we conducted Matrigel-based sprouting assays. The results indicated that they exhibited more extensive matrix sprouting compared to HUVEC spheroids (Fig. S1b, Fig. S2d, e).

To further elucidate the gene expression signature of sarconoids in comparison to their tissues of origin, angiosarcoma tissues and patient-derived sarconoids, collected from consenting patients, were analyzed using RNA-seq (Fig. S3a). A heatmap depicting 33 genes that are upregulated across all four samples is presented in Fig. 1c and Fig. S3e (fold change > 4 and Bonferroni-corrected *p* < 0.001). Furthermore, gene set enrichment analysis techniques, such as gene set variation analysis, were employed to stratify sarcoma into distinct subgroups based on previously reported data [8]. Patient-derived sarconoids were classified into sarcoma, liposarcoma, and chondrosarcoma categories (Fig. 1d). Hierarchical clustering results revealed an enrichment of angiogenesis-associated genes in both tissue and sarconoids derived from angiosarcomas (Fig. S3f). Gene Ontology (GO) term enrichment analysis of the upregulated differentially expressed genes (DEGs) indicated that a variety of DEGs were enriched in biological processes pertinent to angiogenesis, muscle cell differentiation, and extracellular matrix (ECM) organization (Fig. S3b-d). Conversely, GO terms associated with skin development were overrepresented in the downregulated genes (Fig. S3g).

Utilizing UMAP to embed and cluster our single-cell expression data, we identified six distinct cell populations, labeled C0-C5 (Fig. S4a). Each cluster was mapped to compare its most representative expressed genes with known markers (Fig. S4b-d). Utilizing the CellMarker Database, we were able to assign multiple cell type labels to each cluster (Fig. 1e). Notably, Cluster 5, which partially consists of PROCR-expressing progenitor cells, has been associated with vascular endothelial stem cells [9, 10]. PROCR expression in tissue samples was analyzed using both qRT-PCR and immunohistochemistry, revealing high levels in 8 of the 9 angiosarcoma cases as compared to normal tissue (Fig. 1f and Fig. S5b). Additionally, we performed qRT-PCR analyses to assess PROCR expression in patient-derived sarconoids, finding it to be significantly higher in sarconoids from patients #2 and #4 compared to normal cells (Fig. 1g and Fig. S5a).

**Fig. 2:**
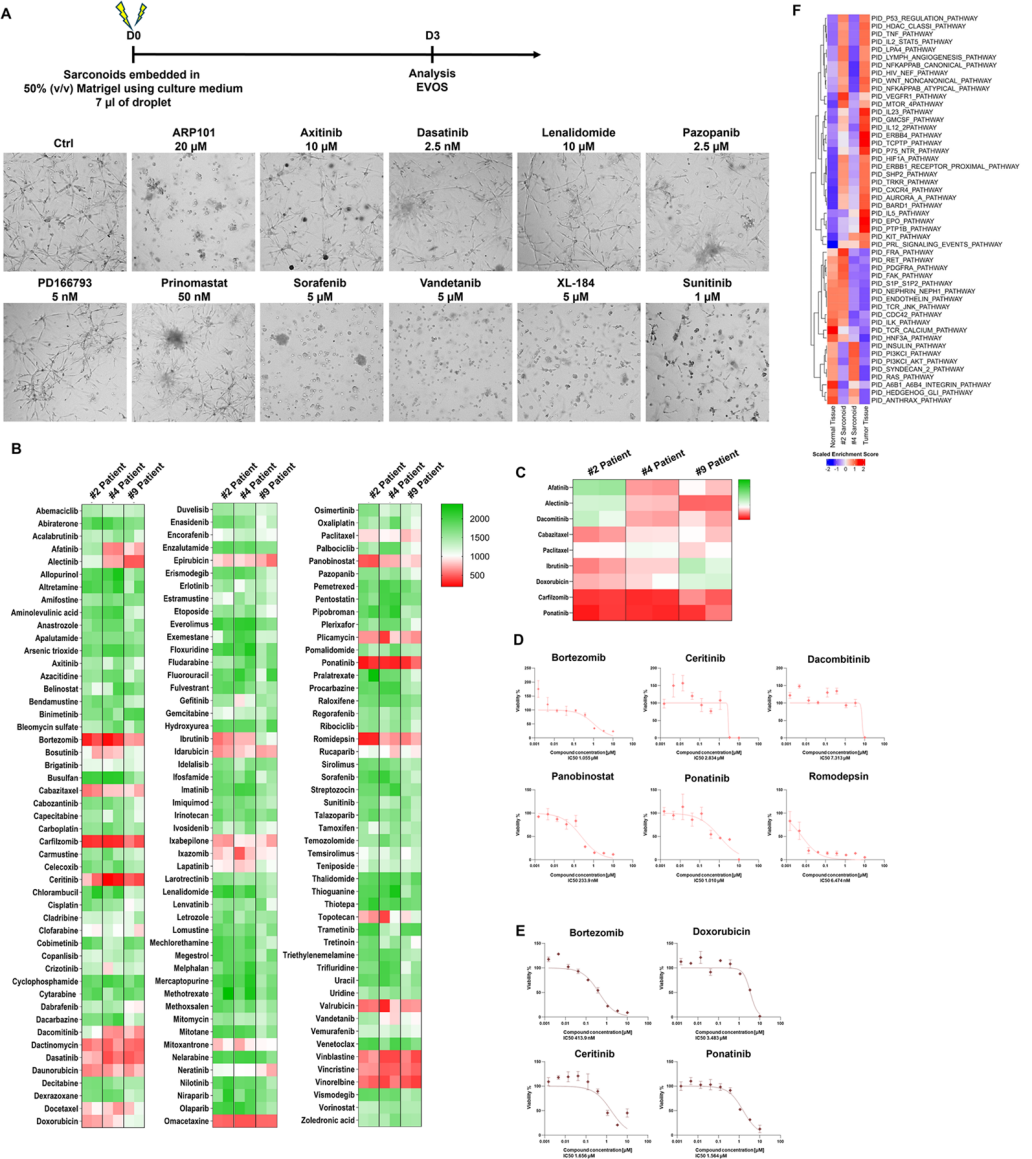
Visualization of high-throughput screening results for 147 FDA-approved compounds against patient-derived sarconoids. (**a**) Experimental design. Representative bright field images show the morphology of the patient #4-sarconoids after three days of treatment with the indicated compounds at different concentrations. (**b**) The 147 FDA-approved compounds were tested in patient #2-, #4- and #9-sarconoids. The percentiles for the obtained anti-proliferative values are depicted using a heat map. Low values (indicating resistance) are depicted in green, and high values (indicating sensitivity) in red. Compounds are ordered alphabetically. (**c**) Heat map of drug effects demonstrating heterogeneous drug responses between the patient #2-, #4-and #9-sarconoids. The columns indicate samples, and the rows indicate the tested drugs and nominal targets. (**d**) Representative drug response curves for bortezomib, ceritinib, dacombitinib, panobinostat, ponatinib, and romidepsin in patient #2-sarconoid. (**e**) Representative drug response curves for bortezomib, doxorubicin, ceritinib, and ponatinib in the patient #4-sarconoids. Normalized mean ± SEM (*n* = 3) viability data from triplicate wells for each drug concentration are presented. (**f**) Sibling cultures from patients #2 and #4 display heterogeneous pathway activity. The HDAC pathway was specifically activated in only the patient #2-sarconoid tissue, which resulted in a better response to HDAC inhibitor compared to patient #4-sarconoids. Only patient #4-sarconoids showed an upregulation of the PI3K/Akt and RAS pathway, which underpinned their better response to the TKIs. The indicated color code represents scaled mRNA expression across the samples

To confirm the suitability of our sarconoid model for drug screening and response prediction, we conducted single-dose trials between 2.5 nM and 20 µM using anti-angiogenic agents on sarconoids derived from patient #4 (Fig. 2a). Using a high-throughput in vitro drug screen, we assessed the cytotoxic activities of 147 FDA-approved compounds on patient-derived sarconoids, observing differing sensitivities between the three sarconoid strains (Fig. 2b). Image-based phenotypic analyses helped categorize individual sarconoids as either insensitive or sensitive to specific treatments (Fig. S6a, b). Overall, 10 compounds demonstrated activity against at least one sarconoid strain. To validate these findings, we performed more comprehensive dose-response assays on 7 active compounds (Fig. 2d, e). Intriguingly, EGFR-TKIs like afatinib and dacomitinib did not significantly impact the viability of sarconoids from patient #2 in our image-based assays, suggesting specific molecular targets and pathways are at play (Fig. 2c). Moreover, sarconoids from patient #2 showed greater sensitivity to histone deacetylase (HDAC) inhibitors like romidepsin and panobinostat (Fig. S6c), suggesting up-regulation of the HDAC pathway in this case (Fig. 2f). Each sarconoid line exhibited unique phenotypic and genomic features, resulting in varied drug sensitivities.

Each sarconoid line exhibited unique phenotypic and genomic features, resulting in varied drug sensitivities. This underscores the importance of tailoring treatments to individual patients, which may help narrow down therapeutic options and optimize the treatment regimen for angiosarcoma.

### Electronic supplementary material

Below is the link to the electronic supplementary material.

Supplementary Material 1

Supplementary Material 2

Supplementary Material 3

Supplementary Material 4

## Data Availability

Any additional information required to reanalyze the data reported in this paper is available from the corresponding authors on reasonable request, gsjeong@amc.seoul.kr.

## Abbreviations

FDA: Food and drug administration
qPCR: Quantitative real-time PCR
HUVEC: Human umbilical vein endothelial cells
RNA-seq: RNA Sequencing
GO: Gene ontology
DEGs: Differentially expressed genes
ECM: Extracellular matrix
UMAP: Uniform manifold approximation and projection
PROCR: Protein C receptor
EGFR: Epidermal growth factor receptor
TKIs: Tyrosine kinase inhibitors
HDAC: Histone deacetylases
HTS: High-throughput screening
