# Supplementary material for: Generation of sarconoids from angiosarcoma patients as a systematic-based rational approach to treatment
Source: J Hematol Oncol. 2024 May 20;17:35. doi: 10.1186/s13045-024-01556-3 (PMC11104004; doi:10.1186/s13045-024-01556-3)
Supplement: Supplementary file 1 — Supplementary Material 1 [file 13045_2024_1556_MOESM1_ESM.docx]

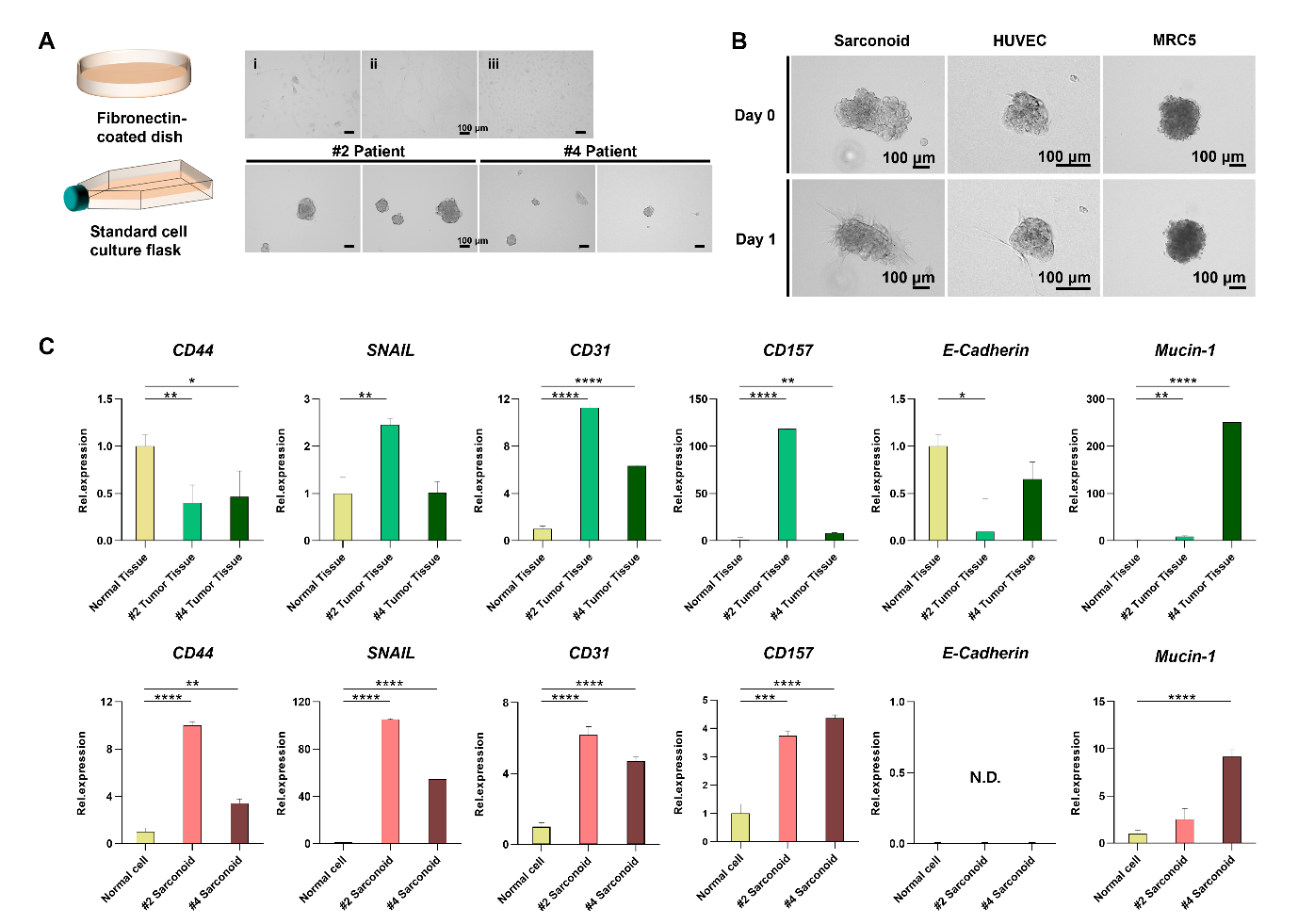


**Fig. S1** Phenotypical characterization of the angiosarcoma patient-derived sarconoids. **a** Representative phase-contrast images of sarconoid formation: i, irregular-shaped morphology; ii, spindle-shaped morphology; iii, cobblestone-shaped morphology. **b** Representative micrographs showing sprout formation in sarconoids, HUVECs, and MRC5 spheroids embedded in Matrigel at 24 hours; scale bar, 100 μm at 10× magnification. **c** Quantitative analysis of relative gene expression levels for mesenchymal (CD44, SNAIL), endothelial (CD31, CD157), and epithelial (E-Cadherin, Mucin-1) markers in normal tissue, tumor tissue from patients #2 and #4, normal cells, and tumor-derived sarconoids, assessed via RT-qPCR (n=3). Statistical significance was evaluated using an unpaired t-test;*p<0.05, **p<0.01, ***p<0.001, and ****p<0.0001.


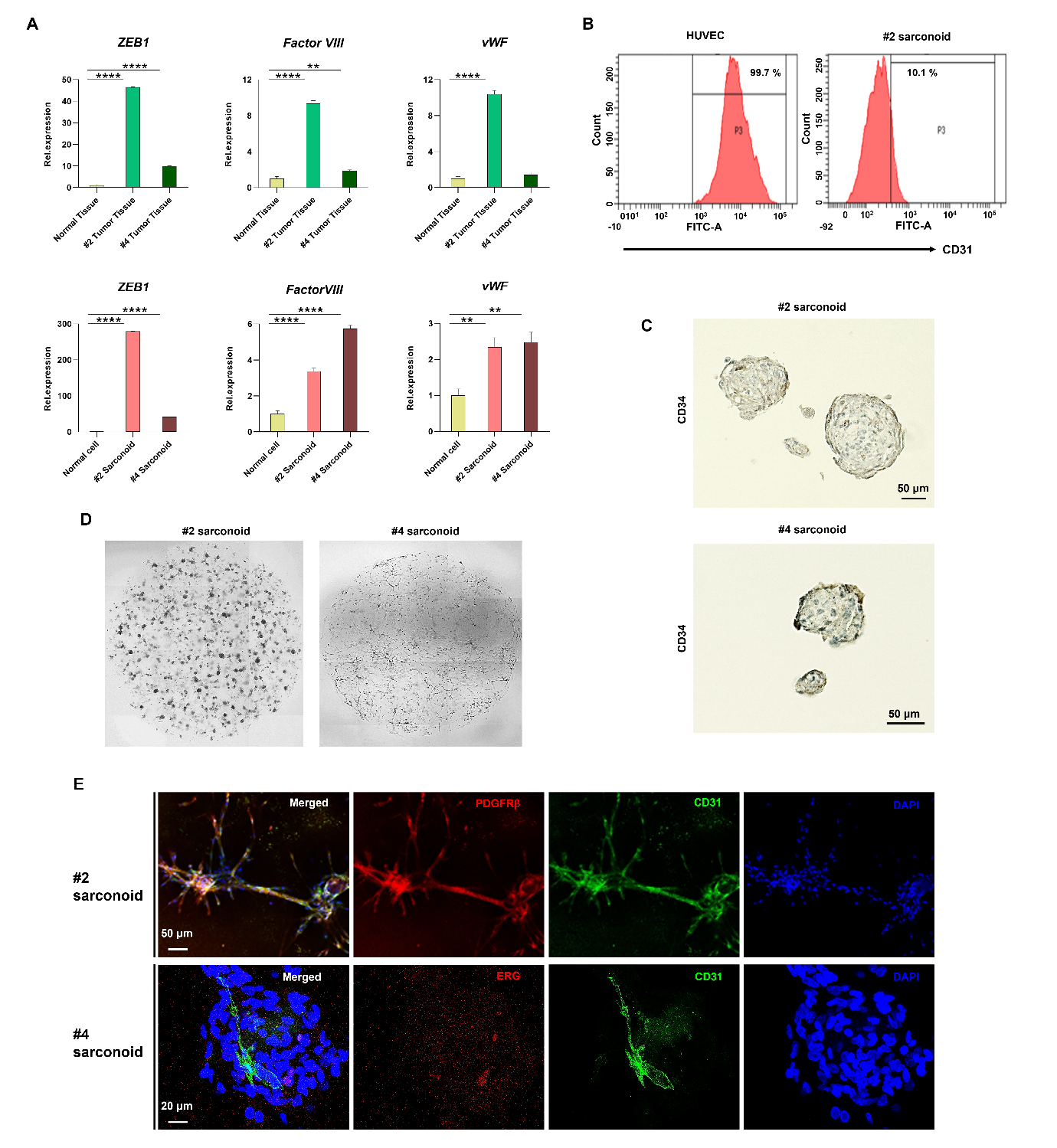


**Fig. S2** Angiosarcoma patient-derived sarconoids exhibited endothelial cell phenotypes. **a** Relative gene expression of mesenchymal (ZEB1) and endothelial (Factor VIII, vWF) markers in normal tissue, patient #2 and #4 tumor tissue, normal cells, and tumor-derived sarconoids assessed by RT-qPCR (n = 3); N.D., not detected. An unpaired t-test was used; *p<0.05, **p<0.01, ***p<0.001, ****p<0.0001. **b** Flow cytometry analysis of CD31 expression in patient #2-sarconoids and HUVECs shown as percentage increase versus isotype controls. **c** Immunohistochemical reactivity of patient-derived sarconoids to CD34. Scale bars, 50 μm. **d** Representative images of the vascular networks from various sarconoids cultured in a 24-well plate at day 1. **e** Immunofluorescence confocal microscopy of endothelial sprouts double-stained for CD31 (green) and PDGFR-b (red; top panel) or ERG (red; bottom panel). Scale bars, 50 μm (top panel) and 20 μm (bottom panel).


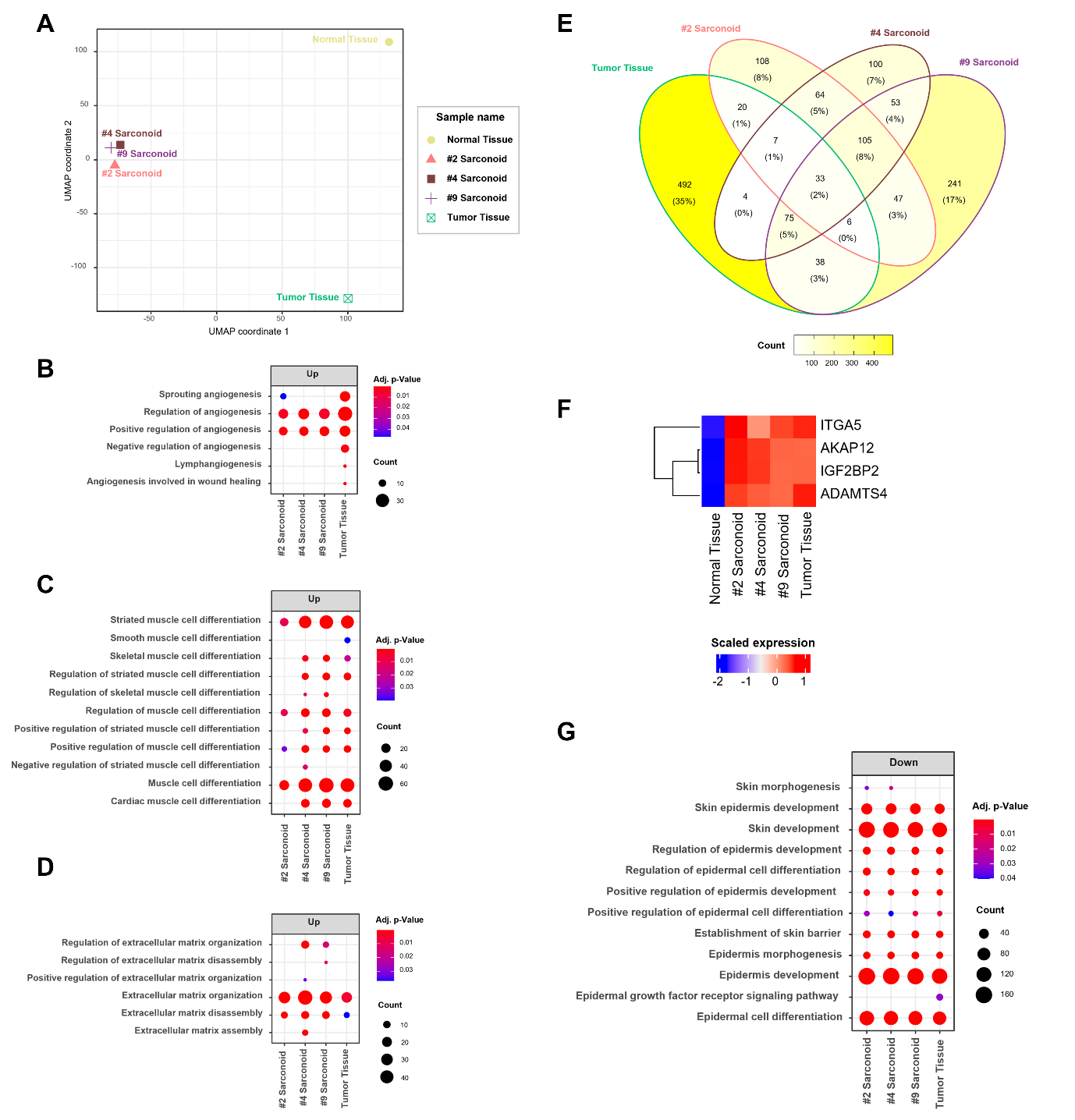


**Fig. S3** Transcriptome profiling of angiosarcoma patient-derived sarconoids. **a** UMAP embedding of RNA-seq data from normal tissue, patient #2 tumor tissue, and patient #2-, #4- and #9-sarconoids. Each data point represents one sample (square, tumor tissue; circle, normal tissue; triangle, patient #2-sarconoid; filled square, patient #4-sarconoid; cross, patient #9-sarconoid). The terms (**b**) angiogenesis, (**c**) muscle cell differentiation, and (**d**) ECM organization were enriched in the patient-derived sarconoids and tumor tissue. The X-axis displays the samples, the Y-axis represents the enriched GOs, the size of the bubbles pertains to the count/number of genes, and the colors refer to the significance (p ≤ 0.001). **e** Venn diagram of the number of upregulated genes commonly or specifically expressed by each sample. **f** Heat map of the expression levels of angiogenesis-related genes increased in patient #2 tumor tissue and patient #2-, #4- and #9-sarconoids compared with normal tissue. **g** Bubble plot of the most significantly enriched GOs in accordance with the most downregulated DEGs of the samples, as indicated. The X-axis displays the samples, the Y-axis represents the enriched GOs, the size of the bubbles pertains to the count/number of genes, and the colors refer to the significance level (p ≤ 0.001).


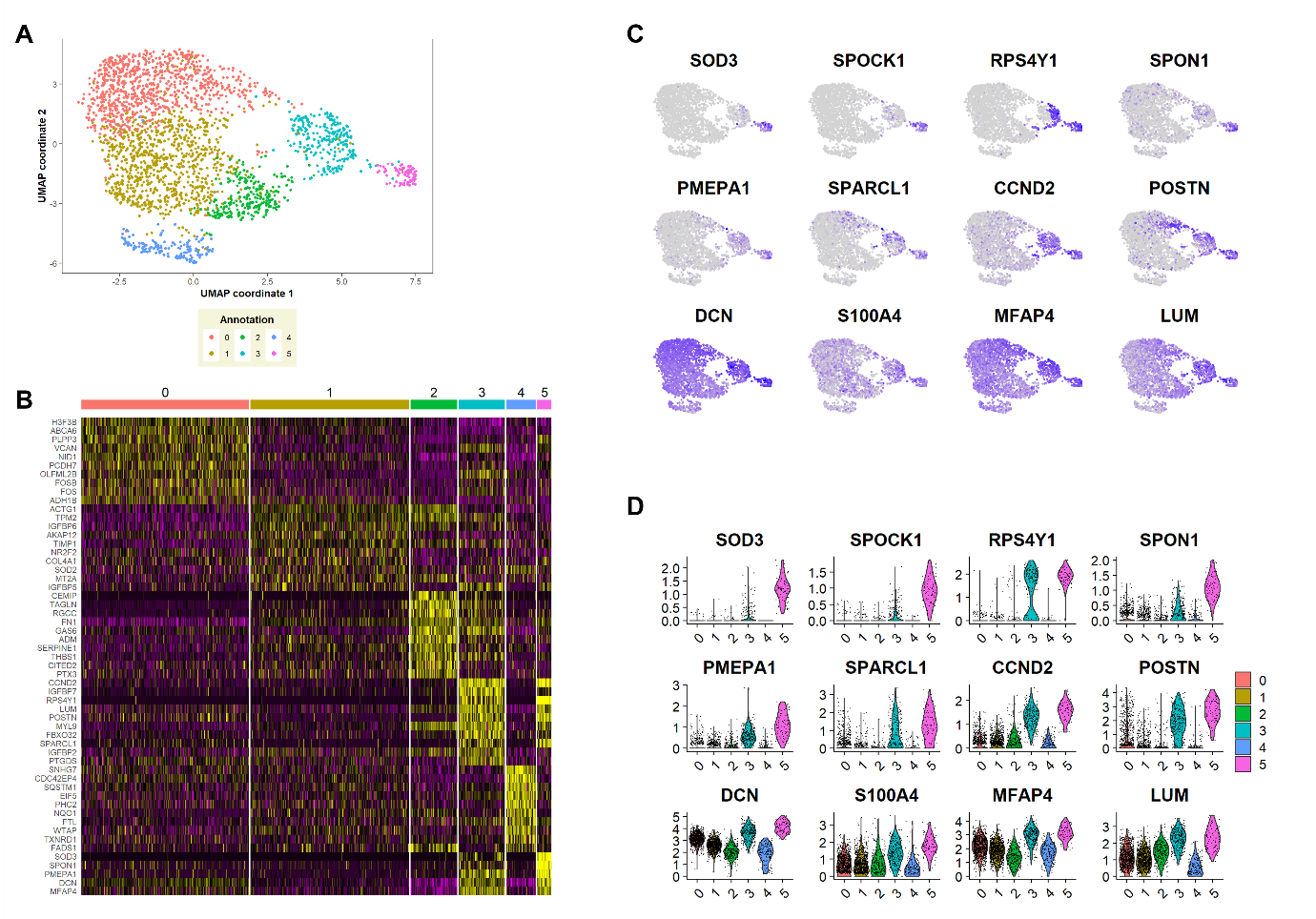


**Fig. S4** Characterizing cellular heterogeneity of patients-derived sarconoids with single-cell resolution. **a** UMAP representation of associated clusters in the patient #2-sarconoids. **b** Gene-expression heat map of the top 10 DEGs across each cluster as compared to all other clusters. Canonical marker identification of distinct clusters in the patient #2-sarconoids. tSNE projections (**c**) and accompanying violin plots (**d**) depict the genes used for the identification.


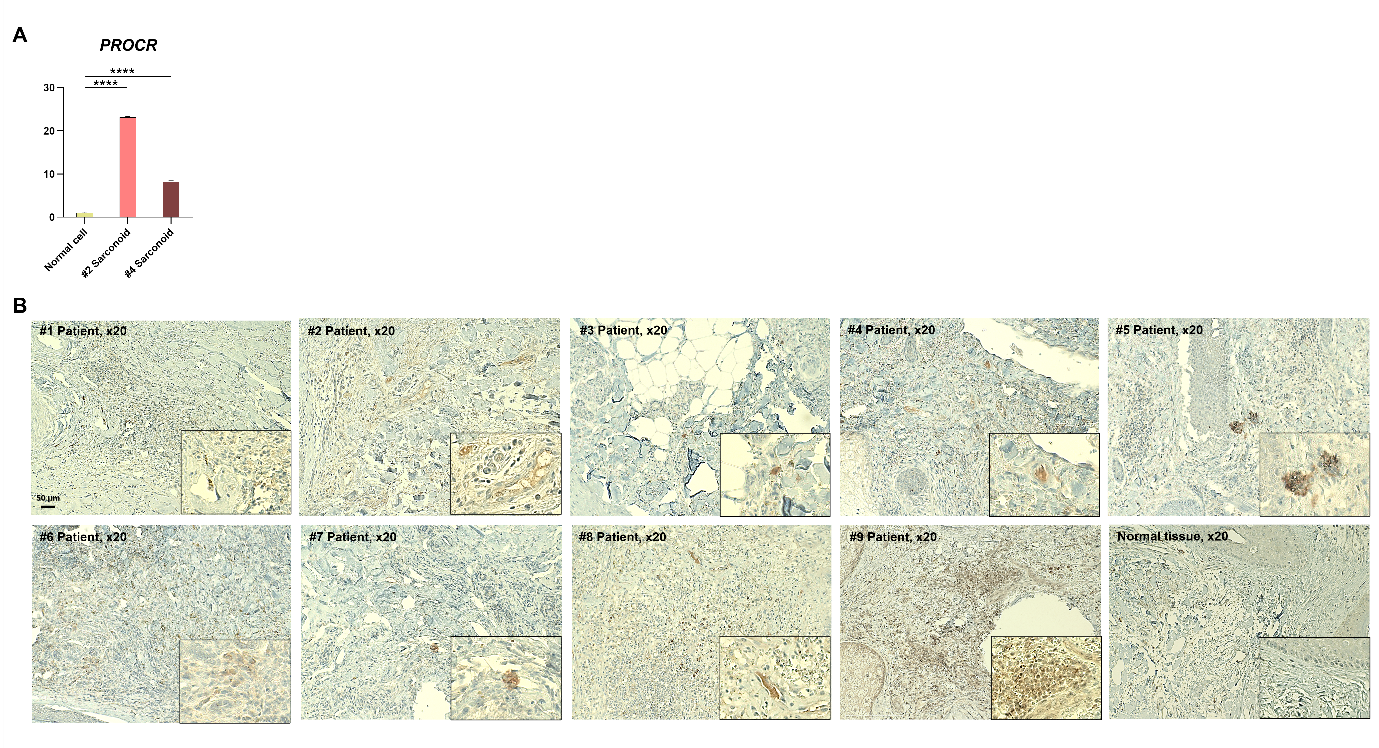


**Fig. S5** PROCR expression levels in angiosarcoma. **a** Expression levels of the PROCR gene in normal cells and tumor-derived sarconoids measured by RT-qPCR. ****p<0.0001, unpaired t-test. **b** Representative immunohistochemical staining images for PROCR in angiosarcoma and normal specimens. Scale bars, 50 μm.


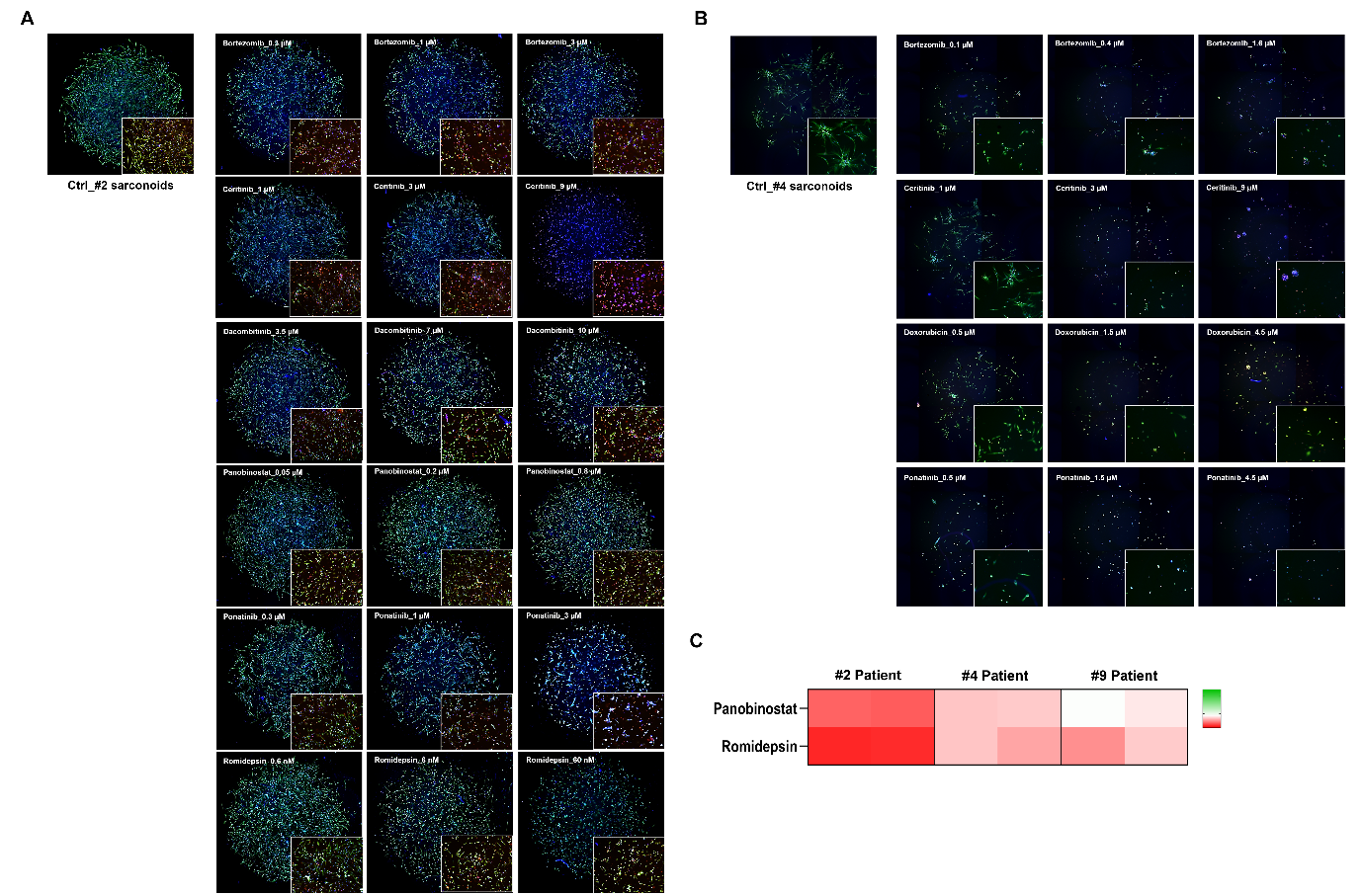


**Fig. S6** Visualization of the drug responses of patient-derived sarconoids. Visual assessment of the dose-dependent loss of elongation and also cell death effects in patient #2- (**a**) and patient #4- (**b**) sarconoids using a live/dead assay. **c** Heat map demonstrating the heterogeneous drug responses between patient #2-, #4-, and #9- sarconoids.
