# Supplementary material for: Generation of sarconoids from angiosarcoma patients as a systematic-based rational approach to treatment
Source: J Hematol Oncol. 2024 May 20;17:35. doi: 10.1186/s13045-024-01556-3 (PMC11104004; doi:10.1186/s13045-024-01556-3)
Supplement: Supplementary file 3 — Supplementary Material 3 [file 13045_2024_1556_MOESM3_ESM.doc]

## **Supplementary Materials and Methods**

**Patient samples.** Angiosarcoma tissue specimens were obtained from nine consented patients who underwent surgical resection at Asan Medical Center in Seoul, South Korea. Ethical approval for the study was granted by the Institutional Review Board of Asan Medical Center. Written informed consent was secured from all participating patients. Detailed information for both cancerous and normal adjacent tissue specimens is listed in Table S1.

**Tissue and sarconoid generation.** Immediately upon collection, tissue samples were stored in MACS® Tissue Storage Solution (Cat#130-100-008; Miltenyi Biotec). Tumour tissues were partitioned into small, approximate fragments and sequentially exposed to 2.5 U/ml of dispase II (Cat#17105041; Gibco™) at 4 °C overnight and 0.04% trypsin-EDTA (Cat#T4049; Sigma-Aldrich) at 37°C for two hours. The digested tissue was mechanically disrupted by vigorous pipetting and subsequently filtered through a 100-µm mesh nylon strainer (Cat#93100; SPL). After centrifugation at 500×g for five minutes at room temperature, the pellet was rinsed with FBS-free endothelial cell basal medium containing a supplemental mix (Cat#1001; ScienCell™).

For the generation of adherent 2D cultures of patient-derived tumour cells, the cells were plated on tissue culture dishes coated with fibronectin (Cat#354403; Corning®) and cultured in FBS-free endothelial cell basal medium, supplemented with B27 without vitamin A (Cat#12587010; Gibco™), N2 supplement (Cat#17502048; Gibco™), 0.1 mg/ml heparin (Cat#H3149; Sigma-Aldrich), and Y-27632 (Cat#1254; TOCRIS). Upon reaching confluence, the cells were passaged using 0.25% trypsin-EDTA (Cat#T4049; Sigma-Aldrich).

For sarconoid production, cells were resuspended at a final concentration of 2 × 10^5^ cells and cultured in uncoated T75 flasks (Cat#70075; SPL). Aggregation into sarconoids was generally observed starting from day 10 of culture, as detailed in Video S1. Sarconoids were harvested every 14 days at the time of medium change and were designated for further analysis. This procedure can be reiterated for multiple cycles. The culture medium consisted of FBS-free endothelial cell basal medium, B27 without vitamin A, N2 supplement, and 0.1 mg/ml heparin.

In some instances, the quantity and quality of the isolated cells did not satisfy the necessary criteria for sarconoid formation, often due to extensive necrotic degradation in the original tissue samples.

**Sprouting angiogenesis assay.** HUVECs and MRC5 were trypsinised and resuspended in EGM2 (Cat#CC-3162; Lonza) or DMEM (Cat#10569010; Gibco™) supplemented with 10% FBS (Cat#16000044; Gibco™), respectively. Methylcellulose (Cat#M0512; Sigma-Aldrich) was added to 0.25%, and 20-µl drops were seeded onto petri dish. To form spheroids, incubate upside-down in a humidified incubator at 37ºC and 5% CO_2_. Spheroids and sarconoids were embedded into Matrigel (Cat#356231; Corning®) and incubated with sprouting promotion medium: EGM2 containing B27 without vitamin A, N2 supplement, 100 ng/ml Recombinant Human VEGF_165_ (Cat#100-20; Peprotech), 0.1 mg/ml heparin, 100 ng/ml FGF2 (Cat#100-18B; Peprotech) and 20 µg/ml Insulin (Cat#17-838Z; Lonza).

**Real-time quantitative PCR.** Total RNAs were extracted from sarconoids and tissues using the mirVana™ miRNA isolation kit (Cat#AM1561; Invitrogen™). Complementary DNA (cDNA) was synthesized from total purified RNA (0.3μg) using a PrimeScript™ 1^st^ strand cDNA Synthesis kit (Cat#611A; TaKaRa). Quantitative PCR was performed with a LightCycler® 480 II system (Roche) using LightCycler® 480 SYBR Green I Master (Cat#04887352001; Roche). RNA isolation, cDNA synthesis, and subsequent quantitative PCR were carried out in accordance with the manufacturer’s recommendations. Relative mRNA expression was calculated using the 2^-ΔΔCt^ method, using GAPDH as the housekeeping gene. All the RT–qPCR primers are listed in Table S2.

**RNA sequencing.** Total RNA was isolated using Trizol reagent (Invitrogen). RNA quality was assessed using a Agilent 2100 bioanalyzer (Agilent Technologies, Amstelveen, The Netherlands), and RNA quantification was performed using ND-2000 Spectrophotometer (Thermo Inc., DE, USA ). Libraries were prepared from total RNA using the NEBNext Ultra II Directional RNA-Seq Kit (NEW ENGLAND BioLabs, Inc., UK). The isolation of mRNA was performed using the Poly(A) RNA Selection Kit (LEXOGEN, Inc., Austria). The isolated mRNAs were used for the cDNA synthesis and shearing, following the manufacturer’s instructions. Indexing was performed using the Illumina indexes 1-12. The enrichment step was carried out by PCR. Subsequently, libraries were checked using the TapeStation HS D1000 Screen Tape (Agilent Technologies, Amstelveen, The Netherlands) to evaluate the mean fragment size.

Quantification was performed using the library quantification kit using a StepOne Real-Time PCR System (Life Technologies, Inc., USA). High-throughput sequencing was performed as paired-end 100 sequencing using NovaSeq 6000 (Illumina, Inc., USA).

A quality control of raw sequencing data was performed using FastQC [1]. Adapter and low quality reads (<Q20) were removed using FASTX_Trimmer [2] and BBMap [3]. The trimmed reads were then mapped to the reference genome using TopHat [4]. Differentially expressed gene (DEG) analysis was performed using EdgeR version 3.36.0 [5] with p values < 0.001 and | Log2(fold change) | > 2 threshold used for significance. Gene ontology (GO) enriched on significant DEGs were accessed using clusterProfiler version 4.2.0 [6]. Enrichment scores for the gene sets in each sample were calculated using GSVA version 1.42.0 [7]. Principal component analysis (PCA) plots, bar plots, box plots, dot plots and heat maps were generated using ggplot2 (version 3.3.5) and ComplexHeatmap (version 2.10.0). All statistical analysis and visualizations were performed under R (version 4.1.1) and R studio envelopment.

**Single-cell RNA-seq.** Single-cell suspensions were washed and resuspended in 0.04% BSA in PBS. Cells were counted automatically with Countess II (Thermo Fisher, Waltham, MA) to determine their concentration. Single-cell RNA-sequencing libraries were the prepared using the Chromium Next GEM Single Cell 3’ reagent kit v3.1 (10X Genomics, Pleasanton, CA) in accordance with the manufacturer’s protocol. Briefly, the cells were diluted into the Chromium Next GEM Chip G as to yield a recovery of ~ 3,000 single-cell. Following the library preparation, the libraries were sequenced in multiplex on the Novaseq 6000 sequencer (Illumina, San Diego, CA) to produce on average a minimum of 60,000 reads per single cell.

**Analysis of single-cell RNA-seq data** Sequencing reads were processed using Cell Ranger version 3.0.1 (10X Genomics, CA) and the Human reference transcriptome GRCh38 from the Ensembl genome database. From the gene expression matrix, downstream analysis was carried out with R version 4.1.1. Quality control, filtering, data clustering and visualization, and differential expression analysis was carried out using Seurat version 4.0.5 R package [8]. For each individual dataset, only the cells with 2,500 ~ 10,000 genes were selected. In addition, we removed any single cell with > 20% UMIs mapped to mitochondrial genes. After normalizing and scaling the data, we performed PCA on the gene expression matrix and used the first 20 principal components for clustering and visualization. Clustering was performed with a resolution of 0.5 and visualization was done using uniform manifold approximation and projection (UMAP). Genes expressed in over 60% of the cells in each cluster and with a binary logarithm of average expression of cells in the cluster over that of cells not in the cluster of greater than 0.15 were selected as cluster markers. For the unbiased annotation of clusters, over-representation analysis [9] of cell types on cluster marker sets was conducted using clusterProfiler version 4.2.0 [6] based on the CellMarker database [10].

**Flow cytometry.** HUVECs and sarconoids were enzymatically dissociated into a single cell suspension and then resuspended in 100 µl Flow Cytometry Staining Buffer (Ca#FC001; R&D Systems™) and stained with FITC Conjugated anti-CD31 antibody (Cat#303103; BioLegend). Flow cytometry was performed using a BD FACSAria II flow cytometer (BD Biosciences).

**Histology and immunohistochemistry.** Tissues and sarconoids were fixed in 10% neutral-buffered formalin solution (Cat#HT501128; Sigma-Aldrich) at room temperature, and then embedded in paraffin. The paraffin-embedded tissues and sarconoids were sectioned at a thickness of 6 µm. After dewaxing and rehydration, the sections were then stained with primary antibodies (Table S3) followed by incubation with a specific horseradish peroxidase (HRP)-labelled secondary antibody, and the positive signals were developed using a diaminobenzidine peroxidase substrate kit (Cat#34002, Thermo Scientific). Haematoxylin and eosin (H&E) counterstaining were carried out in accordance with the manufacturer's instructions (Cat#ab245880; Abcam). The slides were imaged using an EVOS FL Auto 2 microscope (Thermo Fisher Scientific).

**Immunofluorescence double staining for CD31 or PDGFR−β and ERG.** Sarconoids were embedded in Matrigel and overlaid with FBS-free endothelial cell basal medium containing supplement mix, B27 without vitamin A, N2 supplement, 30 ng/ml recombinant human BMP-4 (Cat#120-05ET; Peprotech), 30 ng/ml recombinant human VEGF_165_ (Cat#100-20; Peprotech), 0.1 mg/ml heparin, and 25 ng/ml recombinant human FGF-basic (Cat#100-18B; Peprotech). Sarconoids were treated on day 4 with sprouting promotion medium. After two days of treatment, sarconoids in the Matrigel gels were fixed for 30 min with 4% PFA (Ca#47608; Sigma-Aldrich) at room temperature and PFA was replaced with a permeabilization solution (2% Triton X-100, and 0.2% sodium azide in PBS). Sarconoids were kept at room temperature for two days and then incubated in blocking buffer (10% normal goat serum, 1% Triton X-100, 0.2% sodium azide in PBS) on an orbital shaker at 4 °C for two days. This was followed by incubation with primary antibodies over 2-3 days at 4°C on an orbital shaker at a 1:100 dilution in each case: CD31 (Cat#ab28364; Abcam), PDGFR-β (Cat#AF385; R&D SYSTEMS), and ERG (Cat#MA5-26245; Invitrogen).

The sarconoids were washed in washing buffer (3% NaCl and 0.2% Triton-X 100 in PBS) on an orbital shaker at 4ºC overnight. Samples were incubated with the corresponding secondary antibody on an orbital shaker at 4ºC for 2 to 3 days at a 1:1000 dilution [anti-goat Alexa fluor 647 (Cat#A-21447; Invitrogen), anti-rabbit Alexa fluor 488(Cat#150077; Abcam) or anti-mouse Alexa fluor 647(Cat#A-21235; Invitrogen)]. After overnight washes in washing buffer, samples were counterstained with DAPI (Cat#D1306; Invitrogen) and washed again three times for 30 minutes each in PBS. The sarconoids were cleared in RapiClear® (Cat#RC149001; SUNJin Lab) and were kept in the solution until they were cleared. Confocal images were captured on an LSM 780 microscope (Carl Zeiss, Jena, Germany).

**Drug screening.** Sarconoids cultured in an uncoated T75 flask up to two weeks were harvested and mixed in sarconoid culture medium + Matrigel (1:1 ratio). A 7 μl mixture of sarconoids in each case was then seeded onto 96-well white plates (Cat#655098; Greiner) and incubated at 37 °C with 5% CO_2_ for 30 min to solidify the gel. After gelation, cells were treated with 147 FDA-approved drug compounds at a final concentration of 5 μM. The compound library (AOD IX) was supplied by the NCI's Developmental Therapeutics Program (<https://dtp.cancer.gov/>). Cell viabilities were assessed via a fluorometric (LIVE/DEAD™) and CellTiter-Glo® 3D Cell Viability assay (Cat#G9682; Promega) 72 hours after plating.

**Drug response curve and half maximal inhibitory concentrations.** The selected drugs were prepared as 10-point, 3-fold serial dilutions and tested against **s**arconoids in triplicate starting from a 0.001 μM nominal concentration. Relative cell survival was evaluated 72 hours later using a CellTiter-Glo assay in accordance with the manufacturer’s protocol. IC_50_ values were determined using Graph Pad Prism.

**Live/dead assay.** On day 3 of drug screening, sarconoid viability was determined utilizing the LIVE/DEAD™ Viability/Cytotoxicity Kit (Cat#L3224; Invitrogen) as according the manufacturer's instructions with some modifications. Briefly, the medium was aspirated, and the cells were treated with 2 μM calcein-AM, 4 μM ethidium homodimer-1 and 1μg/ml Hoechst 33342 (Cat#H3570, Invitrogen) diluted in PBS for 15 min at 37°C. This was followed by imaging under the Operetta High Contents Screening system (PerkinElmer) and subsequent analysis using Harmony 3.5.1 high content software (Perkin Elmer).

**Time-lapse imaging.** Time-lapse images were recorded using the Juli live stage (NanoEnTech, Korea). The obtained sequential time lapse images were converted to movie files using Clipchamp.

**Statistical analysis.** All the experiments were performed at least in triplicates. Figure legends describe the statistical test used for each experiment and other parameters associated with the representation of the data.

## **Supplemental References**

1. Simon A: FastQC. Available at: https://www.bioinformatics.babraham.ac.uk/projects/fastqc/. In.; 2010.

2. Lab. H: FASTX toolkit. Available at: http://hannonlab.cshl.edu/fastx_toolkit/. 2014.

3. Bushnell B: BBMap. Available at: https://sourceforge.net/projects/bbmap/. In.; 2014.

4. Trapnell C, Pachter L, Salzberg SL: TopHat: discovering splice junctions with RNA-Seq. Bioinformatics 2009, 25(9):1105-1111.

5. Robinson MD, McCarthy DJ, Smyth GK: edgeR: a Bioconductor package for differential expression analysis of digital gene expression data. Bioinformatics 2010, 26(1):139-140.

6. Wu T, Hu E, Xu S, Chen M, Guo P, Dai Z, Feng T, Zhou L, Tang W, Zhan L et al: clusterProfiler 4.0: A universal enrichment tool for interpreting omics data. Innovation (Camb) 2021, 2(3):100141.

7. Hanzelmann S, Castelo R, Guinney J: GSVA: gene set variation analysis for microarray and RNA-seq data. BMC Bioinformatics 2013, 14:7.

8. Hao Y, Hao S, Andersen-Nissen E, Mauck WM, 3rd, Zheng S, Butler A, Lee MJ, Wilk AJ, Darby C, Zager M et al: Integrated analysis of multimodal single-cell data. Cell 2021, 184(13):3573-3587 e3529.

9. Boyle EI, Weng S, Gollub J, Jin H, Botstein D, Cherry JM, Sherlock G: GO::TermFinder--open source software for accessing Gene Ontology information and finding significantly enriched Gene Ontology terms associated with a list of genes. Bioinformatics 2004, 20(18):3710-3715.

10. Zhang X, Lan Y, Xu J, Quan F, Zhao E, Deng C, Luo T, Xu L, Liao G, Yan M et al: CellMarker: a manually curated resource of cell markers in human and mouse. Nucleic Acids Res 2019, 47(D1):D721-D728.
